# New Roles for Pharmacists in Community Mental Health Care: A Narrative Review

**DOI:** 10.3390/ijerph111010967

**Published:** 2014-10-21

**Authors:** Maria Rubio-Valera, Timothy F. Chen, Claire L. O’Reilly

**Affiliations:** 1 Research and Development Unit, Fundació Sant Joan de Déu, Department of Pharmacology and Therapeutic Chemistry, School of Pharmacy, Universitat de Barcelona, Barcelona 08830, Spain; E-Mail: mrubio@pssjd.org; 2 Faculty of Pharmacy, The University of Sydney, Sydney 2006, Australia; E-Mail: timothy.chen@sydney.edu.au

**Keywords:** pharmacist, mental health care, quality use of medicines, community pharmacy, service implementation

## Abstract

Medicines are a major treatment modality for many mental illnesses, and with the growing burden of mental disorders worldwide pharmacists are ideally positioned to play a greater role in supporting people with a mental illness. This narrative review aims to describe the evidence for pharmacist-delivered services in mental health care and address the barriers and facilitators to increasing the uptake of pharmacist services as part of the broader mental health care team. This narrative review is divided into three main sections: (1) the role of the pharmacist in mental health care in multidisciplinary teams and in supporting early detection of mental illness; (2) the pharmacists’ role in supporting quality use of medicines in medication review, strategies to improve medication adherence and antipsychotic polypharmacy, and shared decision making; and (3) barriers and facilitators to the implementation of mental health pharmacy services with a focus on organizational culture and mental health stigma. In the first section, the review presents new roles for pharmacists within multidisciplinary teams, such as in case conferencing or collaborative drug therapy management; and new roles that would benefit from increased pharmacist involvement, such as the early detection of mental health conditions, development of care plans and follow up of people with mental health problems. The second section describes the impact of medication review services and other pharmacist-led interventions designed to reduce inappropriate use of psychotropic medicines and improve medication adherence. Other new potential roles discussed include the management of antipsychotic polypharmacy and involvement in patient-centered care. Finally, barriers related to pharmacists’ attitudes, stigma and skills in the care of patients with mental health problems and barriers affecting pharmacist-physician collaboration are described, along with strategies to reduce mental health stigma.

## 1. Introduction

There has been an increasing recognition that primary health care practitioners play an important role in the identification, support and management of mental disorders; however the clinical contribution of pharmacists has not been universally accepted or comprehensively defined. Mental, neurological and substance use disorders are estimated to effect more than 450 million people worldwide, yet only a minority receive even basic treatment [1]. Medicines are a major treatment modality of management for many mental illnesses and pharmacists are therefore well positioned to enhance mental health services with the potential to reduce the associated burden of mental disorders. This narrative review aims to describe the evidence for pharmacist-delivered services in mental health care and the barriers and facilitators to increasing the uptake of pharmacist services as part of the broader mental health care team. This narrative review is divided into three main sections: (1) the general role of pharmacists in mental health care; (2) the pharmacists’ role in supporting the quality use of medicines with in medication review, strategies to improve medication adherence and antipsychotic polypharmacy, and shared decision making; and barriers and facilitators to the implementation of mental health pharmacy services with a focus on organizational culture and mental health stigma.

With the aim to provide an evaluation of current knowledge on the role of the pharmacist in mental healthcare, we conducted a narrative review covering a wide range of subjects. The search, although not systematic in nature, included hand searches (expert consultation and review of reference lists of all included citations) and searches in electronic databases (PubMed, Medline, ISI Web of Knowledge and Google Scholar). The databases were searched using terms related to pharmacy and pharmacist interventions and mental disorders. Only papers written in English or Spanish (the native languages of the authors) were included in the synthesis.

## 2. Role of the Pharmacist in Mental Health Care

### 2.1. Pharmacists as Part of Multidisciplinary Teams

In recent decades government agencies and international institutions have supported and encouraged the introduction of pharmacists into multidisciplinary health care teams [2]. This is most evident when financial incentives provided by governments and other third party payers are provided to remunerate pharmacists for the provision of professional services in collaboration with other health care professionals [3]. Commonly cited examples include Home Medicines Review in Australia, Medicines Use Review services in the UK, Medication Therapy Management in the US and medication reviews services in New Zealand [4,5,6,7].

As experts in pharmacotherapy, pharmacists can provide complementary skills, knowledge and attitudes to other health care professionals within a multidisciplinary team context. Specifically pharmacists may contribute to health care teams by detecting and resolving or preventing drug related problems; helping to ensure the safe and efficacious use of medicines; providing comprehensive drug information to patients and other health care professionals; promoting medication adherence; and reinforcing primary prevention and health promotion and lifestyle modification activities in the community.

Pharmacists may work across primary (*i.e.*, community pharmacy or general practice) and secondary care (*i.e.*, specialized care) settings. Pharmacists working in hospital and residential aged care settings may often have access to the patients’ clinical chart and also to established communication channels with other health providers. This greatly facilitates the integration of pharmacists in caring for people with mental disorders in these settings. Some studies point to the positive effects of the integration of pharmacists into the multidisciplinary team caring for people with mental illness [8,9,10,11,12], but the published evidence is limited at present. One study has explored health professionals and consumers attitudes to the role of pharmacists working as collaborative prescribers in mental health in secondary care. Both health professionals and consumers acknowledged the role of pharmacists as collaborative prescribers in mental health, as well as in medication management after assessment and diagnosis by a medical practitioner, and as an integral member of the multidisciplinary team [13].

In contrast, in primary care, pharmacists traditionally have more limited direct access to clinical patient data and other health care professionals, however community-based pharmacists are highly accessible to consumers, as they generally do not operate on an appointment based schedule. This provides consumers with good opportunities to seek advice for the management of minor ailments or preventative care, and sometimes more serious conditions, often before seeking help from their general practitioner (GP), and sometimes in preference to the GP [14]. Hence pharmacists are in an ideal position to provide a first point of contact within the health care system, in a triage-type role or as a link between other health care professionals, especially medical practitioners. This was demonstrated by Wang and colleagues in the US, who evaluated the inclusion of a psychiatric pharmacist in a community health centre where access to a psychiatrist was limited and identified the valuable contribution of the psychiatric pharmacist to act as a link between the different areas of care, such as specialty care, psychotherapy or social security [15]. The collaboration of pharmacists with other health care providers has also shown to have a positive impact in the forensic setting. In one study, monthly meetings between a psychiatrist and pharmacist about the implementation of benzodiazepine guidelines led to a significant reduction in the daily dose of benzodiazepines for those in prison [16].

Another model of collaborative care is the Collaborative Drug Therapy Management (CDTM) in which an agreement is reached between the pharmacists and the physicians so that the pharmacists assume responsibility for completing patient assessments, selecting and adjusting drug regimens and monitoring patients results from pharmacotherapy, among other activities [17]. When integrated with the medical and mental health clinics in a safe setting that served homeless individuals, CDTM has shown to be a good strategy for identifying and addressing medication-related problems [18]. The most frequent interventions in the mental health clinic involved patient education, changes of dosage and restart, change or addition of a medicine.

Case conferencing interventions have also been trialed. A case conference is defined as a multidisciplinary meeting of two or more health professionals to plan care for a specific person with chronic and complex care needs [4]. In the context of medication management review services, case conferencing provides an opportunity for direct face-to-face dialogue between the referring physician and reviewing pharmacist [19]. In an exploratory study investigating the process of decision making in mental health between community pharmacists and physicians, detailed discussion about actual and potential medication-related problems along with strategies to optimize individual medication regimens occurred during face-to-face case conference meetings. Although physicians assumed the final decision making responsibility, they recognized the expertise of the pharmacist especially in relation to improving medication adherence and acknowledged that some patients were more willing to share some information with pharmacists than with the physicians [19].

The available evidence suggests that the inclusion of a pharmacist in multidisciplinary teams can result in significant improvements in pharmacotherapy, which in turn may lead to better overall health care for patients with a mental illness. However, the strategies for increased integration of pharmacists into these teams and their roles within these teams, needs to be further developed and improved in order to achieve a more comprehensive collaborative working relationship.

### 2.2. Pharmacists’ Role in Screening and Risk Assessment in Mental Illness

Depression is the third leading cause of global disease burden yet many people do not seek help for their symptoms [20,21,22]. Mental health stigma has been shown to impact on help-seeking behaviors through both treatment stigma (stigma associated with seeking or receiving mental health care) and internalized stigma (personal shame or embarrassment in seeking help) [23]. In addition, there may be a lack of recognition from consumers on the availability of mental health care services and people may not recognize the symptoms of depression or not relate their symptoms to a depressive illness. Many people with a mental disorder first present to a physician regarding a physical complaint [24]. Fernandez *et al*. found GPs were more likely to make an accurate diagnosis of depression with the patient’s presenting symptoms were mental or emotional in nature, suggesting a need to further educate the public on the core symptoms of depression and how to recognize these symptoms [24].

It has been shown that a simple and cost effective way to increase the early recognition of depression is screening [25]. While routine screening for depression in asymptomatic people remains controversial due to a lack of clinical evidence [26], pharmacists are in a unique position to assist in opportunistic screening for depression in the community setting due to their accessibility (no appointments needed) and the general public’s high level of trust in them [27]. Previous research has concluded that trained pharmacists may be equipped with the skills and knowledge to assist in the identification and support of consumers with a mental illness such as depression [10,28,29,30]. Depression screening and risk assessment has the potential to increase early identification of signs and symptoms of depression, and prompt a referral to appropriate healthcare professional if necessary. The effectiveness of depression screening is predominantly dependent on the screening tool used which takes into account its reliability, validity, feasibility and practicality, and a number of depression screening tools have shown to be reliable and valid in primary care settings such as the Patient Health Questionnaire (PHQ) or the WHO-5 Well-being Index [31,32,33].

Screening and risk assessment services in community pharmacy are common and routinely performed for a range of chronic conditions such as diabetes, hypertension or respiratory disorders [34], yet screening is not commonly performed for depression in the pharmacy setting. A number of recent studies have highlighted the capability of community pharmacists to identify and refer people at risk of depression to appropriate health services and have demonstrated that this is a feasible and effective service for pharmacists to be involved in [35,36,37,38]. These studies identified that pharmacists were able to identify people who were at high risk of depression who had previously gone undetected, screen them for depression and refer on to appropriate health services if required. Notably, some cases involved consumers who were acutely suicidal, highlighting the need for specialized training in mental health first aid which has been shown to improve pharmacy students’ mental health literacy and confidence in handling mental health crisis situations [37,38,39] as well as the need for better pharmacist-physician collaboration that facilitates patient referral to psychiatric services. In recognition of the pharmacist’s role in possible mental health crisis situations the accreditation standards for community pharmacy in Australia now require mental health first aid training for accreditation [40].

A number of barriers and facilitators to the implementation of depression screening services in community pharmacy have been identified [38]. Similar to other research, pharmacists have identified that stigma, both self-stigma and from health professionals, can potentially be a barrier to effective implementation. Other barriers to successful implementation of depression screening services identified included limited time, lack of privacy and inadequate remuneration for the pharmacists’ professional input. An important facilitator identified by pharmacists was raising awareness about community pharmacy being a safe place to discuss mental health issues, via tailored mental health promotions [38]. A recent Australian study highlighted that when consumers developed a trusting relationship with their pharmacist they felt the community pharmacy was a safe place to discuss their mental health concerns, showing the importance of not just the physical space but the rapport with health professionals that is important in mental health care delivery [41].

## 3. Quality Use of Medicines

For many mental disorders such as depression, bipolar disorder and schizophrenia, medicines remain a major modality of treatment. Therefore it logically follows that pharmacists should contribute to the management of mental disorders through the conduct of medication reviews, with the view to achieving the quality use of medicines. Previous literature reviews of the community and hospital settings have highlighted this important clinical role for pharmacists [42,43].

In a review of community pharmacy services for consumers with mental disorders, Bell *et al*. identified 22 community based studies exploring a broad range of professional services offered to consumers with mental disorders (n = 10) and services provided to other health care professionals (n = 12) [43]. The review showed that there was some evidence to support the role of pharmacists in providing medication review services to optimize the use of medicines and reduce the use of potentially inappropriate medicines; and also the benefit of offering medication counselling and treatment monitoring services to improve antidepressant medication adherence. However, it was noted that better designed studies are needed to assess the full impact of professional community pharmacy services in the area of mental health.

A recent review of hospital pharmacy services in mental health care by Richardson *et al*. identified 18 hospital-based studies with interventions spanning medication chart review, assessment of laboratory results and medication prescribing; and in providing education for patients and other health care professionals [42]. Despite the overall conclusion that hospital pharmacists do contribute to inpatient mental health care through the provisions of a range of services, the current level of evidence provided by these studies was limited, when the evidence hierarchy endorsed by the National Health and Medical Research Council (NHMRC) was applied. There were no Level I studies (*i.e.*, systematic review of randomized controlled trials) and only two Level II studies (*i.e.*, randomized controlled trials) with the remaining studies using weaker non-controlled designs (Levels III to IV).

### 3.1. Medication Review

In care homes or residential aged care facilities, the excessive use of psychotropic medicines in the elderly, especially antipsychotic medicines, sedatives and hypnotics, can lead to significant adverse events [44]. In a Cochrane review of eight randomized controlled trials evaluating the optimization of prescribing, generally via medication review, Alldred *et al*., found that interventions were able to identify and resolve medication-related problems and improve medication appropriateness but there was no impact on the a priori primary outcome measures of adverse drug events; hospital admissions; mortality; or secondary outcome measure of quality of life [45]. However when Nishtala *et al*., conducted a more specific review involving a meta-analysis of the impact of medication review and or education within residential aged care facilities on the use of psychotropic medicines, there was statistically significant reduction in the use of hypnotic medicines as determined by a pooled odds ratio of 0.57 (95% CI 0.41–0.79) [46]. However, a similar meta-analysis of the impact of medication review on the use of antipsychotic medicines, which are primarily for the management of the behavioural and psychological symptoms of dementia, failed to reach statistical significance (OR 0.81, 95% CI 0.63–1.04) [46].

Other pharmacist-conducted medication review studies have focused on common adverse effects of psychotropic medicines, in particular anticholinergic and or sedative effects, which can lead to both cognitive and physical impairment [47,48]. Evaluation has centred on the impact of medication review on the Drug Burden Index (DBI), which represents a pharmacologically derived and evidence based tool designed to assess the total anticholinergic and sedative medicine burden of individuals [49]. Two Australian studies have used this tool to examine the impact of pharmacist conducted Residential Medication Management Review (RMMR) and Home Medicine Review (HMR) on the prescribing of medicines with anticholinergic and or sedative properties.

Nishtala *et al*. conducted a retrospective study of 500 RMMRs from 62 aged care facilities and demonstrated that pharmacists conducted medication reviews result in a reduction in median DBI scores from 0.50 to 0.33 post-RMMR [47]. It is known that a one unit increase in DBI has the same effect as three to four additional physical co-morbidities as measured by Health ABC score (HABC), and Digit Symbol Substitution Test (DSST) [50], a well-established measure of cognition influenced by drug use, respectively [49]. Hence this study demonstrated the actual impact of pharmacist conducted RMMRs on significantly reducing exposure of residents to sedative and anticholinergic medicines. Similarly, Castelino *et al*. conducted a retrospective study of 372 HMRs from 155 pharmacists and found that median DBI scores were significantly reduced from 0.50 to 0.22 in community dwelling individuals [48]. Both studies highlight that pharmacist conducted medication reviews can reduce exposure to potentially harmful sedative and anticholinergic medicines, often psychotropic medicines, which in turn may minimise decline in physical and cognitive function.

Over several decades there has been a shift in some countries for mental health services to be delivered within the community setting, where possible, rather than in institutional or hospital based care. For consumers receiving care at a community based mental health setting, Gisev *et al*. used an expert panel, comprising a GP, accredited medication review pharmacist, mental health specialist pharmacist and specialist psychiatrist, to independently evaluate 48 medication reviews conducted by pharmacists. These reviews contained 209 medication review findings and 208 recommendations. The panel members assessed each finding on a five-point scale ranging from strongly agree through to strongly disagree. Panellists strongly agreed or agreed with 76% of the findings and the Kendall coefficient of concordance confirmed this agreement (w score of 0.33, *p* = 0.007). Similarly for medication review recommendations, panelists rated 81% as most appropriate or appropriate, with a w score of 0.41 (*p* < 0.001) confirming agreement. This study highlighted that pharmacist conducted medication reviews for community based mental health consumers results in appropriate findings and recommendations [51]. It is noteworthy that medication review findings and recommendations related to both mental health as well as physical health conditions.

As described above case conferences with pharmacists have proved to have a positive impact in patients on psychotropic medicines. In the study by Schmidt *et al*., monthly multidisciplinary meetings were arranged by pharmacists with nurses, nurse’s assistants and physicians, in which individual medication regimens were discussed. This study showed an increase in acceptable antidepressant, anxiolytic and hypnotic medicine use and a decrease in antipsychotic, non-recommended hypnotic and non-recommended anti-depressant medicines [52]. This improvement in the quality of medicines used was maintained three years after the intervention [53].

### 3.2. Medication Adherence

Lack of adherence to psychotropic medicines remains a highly prevalent problem [54,55,56,57,58,59,60,61,62]. Among these, antidepressants show the greatest rates of non-adherence [63] Treatment adherence to antidepressants is essential in achieving remission, restoring previous levels of functioning and preventing reoccurrence of depression [64,65,66,67] and reduces health care utilization and costs [68,69,70].

A number of studies evaluating the effectiveness of interventions to improve adherence to antidepressants have been conducted [71]. In most cases, these trials considered complex interventions which were multifaceted and involved case management and collaborative care interventions [72]. As pharmacists are directly involved in the dispensing of medicines, they are in a good position to collaborate with patients and support their treatment, assess and promote the importance of medication adherence. In the past ten years a number of attempts have been made to involve pharmacists in the management of patients experiencing depression however the findings from these studies has been mixed [30,73,74,75,76,77,78]. Relatively few studies have demonstrated statistically significant [12,73] or marginally statistically significant improvements in medication adherence [30,74,78]. It is noteworthy, however, that when these data are pooled in meta-analyses, the findings are clearer, with a pooled odds ratio demonstrating a significant benefit from pharmacist interventions in the improvement of medication adherence to antidepressant medicines (OR = 1.64, 95% CI 1.24–2.17) [79]. This effect is in agreement with the results obtained in the improvement of antidepressant use by collaborative care strategies (OR = 1.92, 95% CI 1.54–2.39) [80].

While there have also been efforts to improve adherence to other psychotropic medicines, including antipsychotic medicines for the treatment of psychosis [81], far fewer studies have been conducted. This could be due to the stigma of pharmacists towards more severe mental illnesses [82] or evidence that suggests pharmacists are more comfortable providing services for physical illnesses than they are for mental disorders [82,83,84]. One study evaluating a pharmacy based intervention to improve antipsychotic adherence among patients with a serious mental illness, showed increased medication adherence among the patients that received the intervention [85]. The intervention included the use of unit-of-use packaging, a pharmacist education session, refills reminders and notification of clinicians when patients failed to fill prescriptions.

Despite the evidence pointing to the relevance of the pharmacist in the improvement of adherence to psychotropic medicines, pharmacist interventions to promote adherence are still not common practice. A recent study showed that counseling practices of community pharmacists in response to antidepressant adherence related issues do not always fully align with best evidence and practice [86]. Strategies to improve this include provision of key educational messages including adherence related messages, exploring patients’ concerns, and monitoring medication adherence [86]. In addition, overcoming barriers to implementation of adherence services remain and therefore addressing lack of time and skills, assessment of adherence, transition periods from hospital to the community and conflicts in views between providers that could cause confusion to patients must be addressed [87].

### 3.3. Antipsychotic Polypharmacy

Antipsychotic polypharmacy, the concomitant use of more than one antipsychotic agent, is highly prevalent with rates estimated to be between 20-50% [88,89]. Patients on antipsychotic polypharmacy may experience a higher number of side effects than those on antipsychotic monotherapy, including Parkinsonian side effects, hyperprolactinemia, extrapyramidal symptoms, hypersalivation, sexual dysfunction, sedation/somnolence, cognitive impairment, and diabetes [88]. Antipsychotic polypharmacy has also been associated with increased rates of hospitalization [90,91] and mortality [92].

Many patients on antipsychotic medicines are also using other psychotropic agents, such as mood stabilizers or antidepressant medicines [93], which increases the risk of drug-drug interactions and medication-related problems. Antipsychotic treatment can also interact with non-prescription medicines and complementary and alternative medicines (CAMS) (e.g., St John’s wort) which are frequently used by patients with mental disorders [94]. Prescribers are often unaware of the use of CAMS and non-prescription medicines, as they are often not recorded in the patient’s medical history [95]. This is further complicated when patients visit multiple physicians and obtain prescriptions for potentially interacting medicines not considered originally. In a small scale Australian study of 56 patients in the community mental health setting, pharmacist conducted interviews and medication reviews identified significant rates of psychotropic medicine use outside of doses and combinations usually recommended in evidence based treatment guidelines [96]. For example the dose of antipsychotics was computed to be higher than recommended in national treatment guidelines in almost half of the patient (n = 26, 46%) and the rate of antipsychotic polypharmacy was also judged to be high (n = 23, 41%) [61]. Interestingly, these doses and the rates of polypharmacy were high, even when compared to consumers receiving community treatment orders (CTOs) in Australia [97]. However, it should be noted that the study sample was small and the study did not attempt to evaluate clinical appropriateness of the doses used or the concurrent use of two or more antipsychotic medicines.

### 3.4. Shared Decision Making

Over recent years health care has been evolving from the traditional biomedical model of care towards a more patient-centered care model. Several governmental and nongovernmental initiatives to promote this practice have emerged recently all over the world [98,99]. A patient-centered care model refers to the creation of a partnership relationship between the patients and their families with their health care professionals to evaluate the patient’s needs, values and preferences and adapt care to the patient’s context [100,101]. In mental illnesses, the social and psychological context of the patient is highly relevant and there is a strong movement of patient empowering and participation in health decision making and care [102].

Similarly to other health care professionals, pharmacists also need to be aware of the relevance of the patient-centered care model in their interaction with patients. When pharmacists use a professional communication style, it is associated with better health results [103], higher patient satisfaction with the pharmacist and greater trust [104]. However, it is acknowledged that there remains an opportunity for pharmacists to improve their communication skills with patients [105]. For example, a recent study evaluating the pharmacist-patient interactions in the context of depression consultations showed that the pharmacist adopted a biomedical medication-centered approach when counseling patients [106], with a low proportion of pharmacist communication classified as focusing on psychosocial issues relating to patients or their lifestyles.

Some educational strategies directed to pharmacists in the context of mental health care have been shown to positively impact the pharmacists’ practices and attitudes [29,107,108]. These training programs improved the pharmacists’ confidence in communicating with consumers with a mental illness and their capacity to listen to the patients and to adopt the counseling to their psychosocial context. However, overall, the evidence available suggests a need to improve pharmacists’ awareness of the importance of patient-centred models of health care delivery in mental health. This may include the development of specific training materials and programmes to build the communication skills of pharmacists in the context of mental health.

## 4. Barriers and Facilitators to the Implementation of Mental Health Pharmacy Services

While it is known that there will be barriers to the implementation of any new pharmacy service, there are some particular barriers specific to mental health care that need to be adequately addressed for successful service implementation and progression of the role of the pharmacist in mental health care [109,110].

### 4.1. Organizational Culture

The development of the pharmacists’ role from the preparation and dispensing of medicines to the provision of pharmaceutical care services poses a series of challenges. Several barriers have been identified for the provision of these services [111]. In addition, there are barriers specifically related to mental disorders such as pharmacists’ attitudes, stigma and skills. These barriers may be related to the organizational culture of the pharmacy [112]. Organizational culture has a great impact on the provision of care by mental health providers, and in addition individual pharmacist’s beliefs, values and attitudes are a key aspect of organizational culture and can act as a barrier for the provision of these services [110,113,114].

However, organizational culture in pharmacy has not been comprehensively studied [115] and its impact on pharmaceutical services addressed to people with mental disorders has not been assessed. The influence of organizational culture on the adoption of new mental health care roles by non-medical primary health and social care services was recently explored [116]. Organizational culture predicted involvement in mental health promotion and evaluation and education at a system level (*i.e.*, working with other services to provide integrated health promotion programs that include social and emotional wellbeing; evaluating the effectiveness of services for people with, or at risk of depression; and educating the public about how to maximize mental health and emotional wellbeing).

A review of literature relating to organizational culture in community pharmacy practice identified the business *vs*. professional role dichotomy as a key dimension of organizational culture in pharmacy [115], given that the remuneration of pharmacies is predominantly linked to the supply of medication. At times this business role may conflict with pharmacy services focused on the improvement of the effectiveness and quality of the patients’ treatment, increasing the business-professional role conflict. Positively, the model of remuneration has been adapted in some countries to facilitate the implementation of pharmaceutical care services, but this is not widespread nor is it the major source of remuneration for pharmacists. Other structural changes necessary to facilitate the implementation of these services are related to the reconfiguration of the pharmacy to incorporate private consulting rooms, further use of technology and collaboration with other health care providers [110].

### 4.2. Mental Health Stigma 

Stigma associated with mental illness is defined as being a negative attitude, based on prejudice and misinformation, that is triggered by a marker of illness [117]. It consists of three elements; problems of knowledge (ignorance), problems of attitude (prejudice) and problems of behavior (discrimination) [118]. A growing body of evidence suggests that mental health professionals can also be a source of stigmatizing attitudes and behaviors [119,120,121,122]. Stigma can also be manifested through diagnostic overshadowing where individuals are treated differently by physicians once their mental health diagnosis is revealed [123,124]. In particular, literature suggests that consumers report mental health staff to be one of the most stigmatizing of all groups [119,125]. This has significant implications which include: social marginalization which reduces help seeking and leads to undertreatment; lack of access to care; and non-adherence to treatments [126,127]. Further, it is known that those with mental illness have poorer physical health with an estimated 15–20 year lower life expectancy when compared to the general population [128].

Independent of how well health professionals recognize mental illnesses or how knowledgeable they are about treatments and causes for mental illnesses, they may have as many negative stereotypes as the general public [129,130,131]. Consequently, mental health professionals including pharmacists have a crucial role in directing health outcomes, as they can act to both increase or reduce stigma [132]. While some studies have found pharmacists to have generally favorable attitudes towards people with depression and mental illness [83,133,134,135], pharmacists have also reported more stigmatizing views towards people with schizophrenia than depression [84]. This is consistent with international data measuring the stigma and attitudes of pharmacy students which show suboptimal attitudes are present in this cohort towards people with schizophrenia and depression [136,137,138,139,140]. Furthermore, pharmacists have reported a higher level of comfort in discussing medication use in depression than schizophrenia [141], being uncomfortable discussing symptoms of mental disorders and felt they were less likely to follow up consumers with a mental illness than people with a cardiovascular illness [133,141]. Lower levels of mental health stigma have also been shown to be linked with pharmacists’ willingness to provide professional pharmacy services to consumers with schizophrenia [82].

### 4.3. Pharmacists’ Education and Training to Reduce Mental Health Stigma

Suboptimal attitudes towards mental illness and a lack of confidence to provide pharmacy services to mental health consumers highlights the need for different educational approached in the mental health field. Research has identified the need for educational programs to move from the traditional focus of pharmacology and therapeutics of psychotropic medicines and adopt evidence based approaches to reduce mental health stigma and improve pharmacists’ confidence in providing mental health services [107,136,142,143].

While there are a variety of types of anti-stigma interventions that have shown to have evidence for effectiveness contact-based interventions, facilitating personal contact with people with a mental illness as an approach to stigma reduction [143,144], have been found to be more likely to be successful [145,146,147].

Contact-based educational interventions are based on the work of Allport which states that simple contact between groups does not necessarily engender improved intergroup relations, but certain conditions of the contact are required for stigma reduction to be facilitated [148,149]. Allport’s theory of intergroup contact suggests certain prerequisite conditions for the contact to be successful in reducing prejudice. These include equal status between groups or participants, common goals for the interaction, intergroup co-operation and support of the authorities, law or custom [145,148,149,150,151,152]. Many of the required conditions can be integrated into pharmacist and pharmacy student training, such as the condition of equal status. By incorporating mental health consumers as educators in training programs, they can be elevated in status from a standard patient likely to be encountered in a pharmacy setting, which may give them a greater sense of equality. This has been shown in mental health contact based studies with pharmacy, nursing and medical students where the consumers are on equal status with the students and have common goals for the interaction [107,108,153,154,155,156]. Fostering opportunities for students to experience contact in a safe, non-confrontational educational setting has proven to be an effective method at reducing stigma and hence improving their willingness to provide health care services to mental health consumers as pharmacists [82]. Thus educational programs need to be multifaceted and involve contact with mental health consumers as a core element to successfully address mental health stigma and to allow pharmacists to take on a more significant role in the care of mental health consumers [39,107,108,153]. While there is good evidence of the benefits of contact based education in pharmacy and medical students, there is a lack of data on effective interventions to reduce stigma among healthcare staff and it not clear if the evidence base at the tertiary student level is transferable to health professionals in practice. Further research is required to evaluate mental health training programs for pharmacists at all levels.

### 4.4. Factors Affecting Pharmacist-Physician Collaboration

Despite these examples of multidisciplinary care and the relative expertise in pharmacotherapy, pharmacists are often overlooked as core members of health care teams. The integration of pharmacists into health care teams is a constant challenge and remains a core aspect of pharmacy practice based research [30,157,158,159,160]. Several models have been developed which attempt to describe and contextualize the complex relationship in multidisciplinary health care teams, but few specifically focus on the integration of pharmacists in these multidisciplinary teams. Two of these models are the Bradley [158] and the McDonough models [161]. Both models classify pharmacist-physician collaboration in stages that go from professionals’ isolation to a stage of full collaboration. The models identify a series of factors affecting or characterizing the different stages of collaboration (*i.e.*, characteristics of participants, context and exchange). Other related research has focused on the broad topic of implementing new professional pharmacy services. Qualitative studies have been conducted to explore the barriers and facilitators to collaboration according to pharmacists and/or physicians [109,110,111,162]. Key factors affecting collaboration were related to perception of usefulness of collaboration, mutual knowledge and trust, role definition, pharmacists’ conflict of interest and the physicians’ territoriality and hierarchy [30,157,158,163,164]. Models of interprofessional care such as the ones described above take into account the barriers and facilitators for collaboration, and constitute a tool to design new strategies to include pharmacists as part of multidisciplinary teams.

The barriers for pharmacist-physician collaboration could be interpreted within the framework of Ajzen’s theory of planned behavior [165]. This is a model for the prediction of behavioral intention (*i.e.*, pharmacists and physicians’ intention to collaborate). The main components affecting behavioral intention are attitudes toward the behavior, subjective norms and perceived behavioral control. It has been documented that that physician and pharmacist intention to collaborate is strongly linked to their attitudes toward collaboration which in turn, is known to be affected by their perception of the usefulness of the collaboration, mutual trust and knowledge [30,158,163,164].

Negative and neutral opinions about collaboration prevent practitioners from initiating collaboration with the other health professional groups [164]. However, this is usually found between professionals that have not previously experienced collaboration [163,164]. Hence the importance of joint interprofessional continuing education and face-to-face meetings at the practice level. Such interventions may provide a positive interprofessional experience for pharmacists and physicians which helps to reduce conflict and facilitate improved knowledge and understanding about professional roles, increased interprofessional dialogue, shared goals and the promotion of trust [30,157,158,160,163,166]. Other external factors may also facilitate the extent of collaboration [30,158,163]. These include geographical proximity (as opposed to isolation) of pharmacists and physicians; the imprimatur of professional and or government organizations to foster collaboration; legislative factors such as a requirement to share of patient records and economic facilitators such as remuneration for professional services [30,158,163]. Consideration of the aforementioned factors is important when designing new models of care which involve the integration of pharmacists into multidisciplinary teams aimed at providing comprehensive mental health care.

## 5. Conclusions 

This narrative review has demonstrated that pharmacists have a broad range of skills in medication management, provision of drug information to prescribers, counseling patients about medicines, and facilitating medication adherence strategies in the delivery of mental health care. However, many of the published studies evaluating these professional roles for pharmacists have not utilized controlled designs. Hence it is recommended that future evaluation studies include study designs which provide direct evidence for the efficacy of these pharmacist services. The preliminary evidence supporting the role for pharmacists in services such as collaborative drug therapy management, participation in multidisciplinary teams, and in reducing antipsychotic polypharmacy is positive, but future studies should use more robust study designs, more specific and sensitive evaluation measures and involve longer follow up periods. Similarly, studies which develop and evaluate multi-faceted and patient tailored pharmacist services designed to improve medication adherence, would help build on the existing evidence. Finally, understanding and overcoming barriers to the widespread uptake of evidence-based pharmacy services may require specific strategies and training approaches which include mental health stigma reduction.
